# 

**Published:** 1888-01

**Authors:** 


Vol. XLII. JANUARY, 1888. No. 1.

LIBRARY
INDEXED
S.G.O.

### THE DENTAL REGISTER

A MONTHLY JOURNAL.

DEVOTED TO THE INTERESTS OF THE PROFESSION.

EDITED BY

J. TAFT, D.D.S.

PUBLISHED BY
SAM'L A. CROCKER & CO.,
SUCCESSORS TO SPENCER & CROCKER,
OHIO DENTAL AND SURGICAL DEPOT,
Nos. 117, 119, 121 WEST FIFTH STREET, CINCINNATI, OHIO.

**TERMS:—$2.00 per Annum, in Advance. Single Copies, 25 cts.**

